# Health facility or home delivery? Factors influencing the choice of delivery place among mothers living in rural communities of Eritrea

**DOI:** 10.1186/s41043-018-0153-1

**Published:** 2018-10-22

**Authors:** Meron Mehari Kifle, Hana Fesehaye Kesete, Hermon Tekeste Gaim, Goitu Seltene Angosom, Michael Berhane Araya

**Affiliations:** 1 Department of Epidemiology and Biostatistics, School of Public Health, Asmara College of Health Sciences, Asmara, Eritrea; 2 Ministry of Health, Asmara, Eritrea

**Keywords:** Antenatal care, Health facility delivery, Home delivery, Rural community, Eritrea

## Abstract

**Background:**

In Eritrea, despite high antenatal care (ANC) use, utilization of health facilities for child birth is still low and with marked variations between urban and rural areas. Understanding the reasons behind the poor use of these services in a rural setting is important to design targeted strategies and address the challenge contextually. This study aimed to determine factors that influence women’s choice of delivery place in selected rural communities in Eritrea.

**Methods:**

A cross-sectional survey of 309 women aged 15–49 years with a delivery in the last 1–2 years prior to the survey was conducted in a randomly selected villages of Hadish Adi, Serea, Genseba, Kelay Bealtat, Dirko, Mai Leham, Kudo Abour, Adi Koho, and Leayten. Data were collected using an interviewer administered questionnaire. Chi-square tests were used to explore association between variables. Using odds ratios with 95% confidence intervals with *p* < 0.05 taken as statically significant association, bivariate and multivariate logistic regression analysis were used to identify factors that affect the choice of delivery place.

**Results:**

Overall, 75.4% of the respondents delivered their last child at home while 24.6% delivered in health facility. Women whose husband’s had no formal education were less likely [AOR = 0.02; 95% CI 0.01–0.54] to deliver in health facility. Women who had joint decision-making with husbands on delivery place [AOR = 5.42; 95% CI 1.78–16.49] and women whose husbands choose health facility delivery [AOR = 2.32; 95% CI 1.24–5.11] were more likely to have health facility delivery. Respondents who had medium wealth status [AOR = 3.78; 95% CI 1.38–10.37] have access to health facility within 2 km distance [AOR = 14.67; 95% CI 2.30–93.45] and women with traditional means of transport [AOR = 9.78; 95% CI 1.23–77.26] were also more likely to deliver in health facility. Women who read newspaper daily or infrequently had three [AOR = 3.77; 95% CI 1.12–4.04] and almost three times [AOR = 2.95; 95% CI 1.01–8.59] higher odds of delivering in health facility. Similarly, women who have knowledge about complications during delivery [AOR = 4.39; 95% CI 1.63–11.83], good perception on the quality of care they received [AOR = 9.52; 95% CI 1.91–47.50], had previous facility delivery [AOR = 2.69; 95% CI 0.94–7.68], have negative experiences of delivery outcomes in her community [AOR = 1.31; 95% CI 1.00–4.96], and women who perceive home delivery as life threatening [AOR = 1.84; 95% CI 1.46–3.38] were more likely to deliver in health facility.

**Conclusion:**

To increase health facility delivery, raising women’s awareness on the benefits of delivering in health facility, male involvement in the use of maternal health services, increasing women decision-making power, addressing common barriers of lack of transport, and compensations for transport expenses to alleviate the cost of transport are recommended. Efforts to shorten distance to reach health facility and health education focusing on the potential threats of delivering at home at the individual and community level can have substantial contribution to increase health facility delivery in rural communities of Eritrea.

## Background

Over the past 25 years, the global maternal mortality ratio (MMR) has fallen by nearly 44% from an estimated 385 maternal deaths per 100,000 live births in 1990 to 216 in 2015. Yet, this decline has been highly disproportionate, with low-income countries carrying the largest burden. In 2015, low-income countries accounted nearly all (99%) of the global maternal deaths, with sub-Saharan Africa alone accounting for roughly 66% of the death toll [1]. This situation is particularly critical in the Sub-Saharan Africa (SSA) region, where high fertility rates, high lifetime risk of maternal mortality, weakened health systems, poor health-seeking behavior, and poverty have led to decades of stagnation on maternal mortality reduction rate [2–5].

Most incidents of maternal deaths are due to direct obstetric causes such as hemorrhage, sepsis, unsafe abortion, obstructed labor, and hypertensive disorders [6]. These complications occur around the time of delivery and are difficult to predict, but can be effectively managed and deaths averted through health facility delivery equipped with skilled birth attendants placed in an enabling environment [7]. Skilled attendants can perform deliveries either at home or health facilities, but the most efficient strategy for lower income countries like Eritrea is to place them in health facilities with a reliable referral system [8]. The World Health Organization (WHO) recommends that every delivery be overseen by a skilled birth attendant (SBA)—a health professional who can identify and manage normal labor and delivery; and identify and treat complications or provide basic care and referral [9, 10]. However, the proportion of deliveries by SBAs is still below the recommended levels. In Sub-Saharan Africa, about half of births are assisted by SBAs. Even in countries where antenatal care (ANC) is common, a large proportion of deliveries occur at home [11–13].

The choice of delivery place has consistently been found to be associated with maternal and neonatal outcomes. Childbirth in a health facility attended by skilled birth attendant is associated with lower rates of maternal morbidity and mortality than home births [14–16]. Delivery in health facility also plays a critical role in preventing still births and improving newborn survival [7, 17]. Given the demonstrated health benefits of institutional deliveries, it is necessary to understand the range of factors associated with the choice of delivery place. Studies of health care use have highlighted a range of potential influences on a woman’s tendency to seek care. Demographic factors that have been shown to increase the likelihood of health service use are younger maternal age, marital status, low parities, high level of autonomy, being employed, use of modern contraceptives, facility use in the previous delivery, antenatal care utilization, past history of obstetric complications, perceived high quality of care, and high level of husband’s education [7, 18–25]. More than demographic factors, socioeconomic factors appear to be more important determinants of health service use. The most consistently found determinant of use of reproductive health services is woman’s level of education [7, 26]. Cost of care seeking for transport, medications, and opportunity costs of travel time has often been shown to be a barrier to service use [24]. Socioeconomic indicators such as urban residence [27, 28], household living conditions [29], household income [30], and occupational status [31] have also proven to be strong predictors of a woman’s likelihood of using reproductive health services. Besides demographic and socioeconomic factors, the individual environment including community beliefs and norms relating to health behaviors pose a strong influence on the use of health services. Studies have shown that both demographic and socioeconomic determinants of reproductive health services are mediated by community influences on health-seeking behavior that shape the way individuals perceive their own health and the health services available [18, 32]. These community beliefs and norms are reflected in an individual’s health decisions because behavior is influenced by how a person thinks the community views his or her actions. As such, women make delivery decisions within a community and national context. Little is known about the interplay between national level systemic factors and individuals’ delivery choices. However, some studies posit that in low- and middle-income countries, the macro social factors, particularly health system characteristics like health worker to population density, higher national income, urbanization, and lower income inequality affect utilization of facility delivery and may be more efficient in reducing maternal mortality than are interventions aimed at individual women [33–35].

Located in the Horn of Africa, Eritrea gained independence in 1991. At that time, the overall health condition in general and maternal health indicators in particular were very poor in the country. Critical shortage of health services, inadequate number of health care providers, poor health-seeking behavior, deeply rooted harmful traditional practices like female genital mutilation, domestic violence, early marriage and childbirth, low levels of education, low contraceptive use, and traditional gender roles which limit women autonomy and decision-making power were widespread throughout the country [36]. The maternal mortality ratio was as high as 998 per 100,000 live births, one of the highest figures in Africa. The proportion of women delivering in health facility was very low mainly due to long distance to the already scarcely distributed health facilities and the significant cost of transport expenses. To avoid such barriers, many women used to seek delivery services from traditional birth attendants, who were locally available and provided services for free but posed a threat as they practiced unsafe delivery practices and were unable to manage most of the commonly seen complications that occur during delivery.

Since independence, the government of Eritrea through the Ministry of Health has been working tirelessly to ensure the provision of essential health care services and increased accessibility to those services in equitable and affordable means. But shortly after independence, Eritrea was drawn into a border dispute with Ethiopia, and the subsequent war has severely impacted the economy, disrupted the population dynamics and affected all development aspects of the country including the health sector. With a population size of about four million, Eritrea currently stands with the lowest per capita income countries. The total fertility rate is high (an average of 4.5 children per woman) with only 19.6% of women who have their need for family planning satisfied with modern methods [37].

In Eritrea, even though antenatal care (ANC) use is relatively high, the utilization of health facilities for child birth is still low. In 2015, the coverage of at least one prenatal care was more than 95% and about 80% of urban women had four plus antenatal visits, while only 47% of their rural counterparts had four plus visits. Even more pressing challenge is that there are marked variations between urban and rural areas in the proportion of births delivered in health facilities. For instance, the Eritrean Population and Health Survey (EPHS) 2010 shows that only 17% of rural women delivered in health facilities, as compared to 63% in other towns, and 93% of mothers who reside in the capital city. The most commonly cited reasons or barriers for not seeking delivery service were getting money for treatment (39%), having to take transport (35%), and distance to health facility (33%). Around 23% of the women responded that they did not want to go alone, and 20% cited queuing for treatment as a barrier to seeking care. About 12% of respondents also stated that poor quality of care was barrier as well. Some women admitted that they did not know where to go, and 11% mentioned getting permission from husband as a barrier to seek delivery service. Regardless of the number of living children, marital status, or employment status, all respondents in the survey identified getting money for treatment as the main barrier to accessing healthcare. More respondents in the youngest age group (15–19), from rural areas, with no education and from the lowest and second wealth quintile reported having to take transport and distance as the main barriers to accessing health care [38]. Despite these challenges, Eritrea has made a considerable progress in reducing maternal mortality rate during the last two and half decades. Recently, the 2017 national lot quality assurance survey (LQAS) reported that about 96% of pregnant women received antenatal care (ANC) while 62% of childbirths occurred in health facilities [39]. The MMR showed a 68.5% change reduction between 1990 and 2015, with 4.6% average annual decrease [40]. According to the second Health Sector Strategic Development Plan (HSSDP), these trends are likely to continue with increased political will and commitment and health sector spending in the next 5 years [41]. These improvements have been largely due to emphasizing on the functional components of essential maternal health services (quality ANC, health facility delivery, emergency obstetric care, family planning, and postpartum care) and centralization of obstetric services in high density population areas [42]. Moreover, the effectiveness of a community-based preventive interventions has been visible. For instance, Turan et al. found that a low-cost, community-based intervention in Eritrea was associated with not only of significant improvements in safe motherhood knowledge but also an actual use of essential maternity services, including health facility delivery [43].

In most cultures of Eritrea, a large family is traditionally considered a source of higher status, security, and insurance to the family members. Underscoring the desire of childbearing in Eritrean cultures, some studies indicate that daily tasks of childcare and childbearing are a source of deep satisfaction among Eritrean women [44]. Most rural societies still exist as networks of mutually interrelated and dependent groups, emphasizing family or group rather than self and the individual [36]. In this context, women living in the rural areas of Eritrea are less likely to make individual reproductive decisions independent from those of their husband, family networks, or the community they live in. In short, reproductive preferences are issues that go beyond the individual women, being affected by many community factors including culture, religion, and family. Therefore, this study aimed to assess the range of factors associated with the preference of women on delivery place in selected rural communities of Eritrea.

## Methods

### Study setting

The study was conducted from July 2017 to February 2018 in Debub and Northern Red Sea (NRS) zones. In Zoba Debub, two subzones were selected, namely, Emni Hayli and Mai Aini. Emni Hayli is 30 km from Mendefera, the capital city of Zoba Debub. With a total population of 66,350, the subzone has 12,670 women of reproductive age (15–49 years). Commonly seen economic activities of the subzone are rainfall-dependent agricultural activities and livestock rearing. The subzone has one health center and two health stations with roads that are difficult to use throughout the year. Mai Aini is 80 km from Asmara, the capital city of Eritrea. With a total population size of 53,194, about 10,639 of the population are women of reproductive age. The area enjoys fair amount rainfall during rainy seasons. Mai Aini subzone has one community hospital, one health center, and one health station. In northern red sea zone, Leayten administration unit was selected. The area has a total of 400 inhabitants. The region’s ethnic composition is varied. The majority of inhabitants are Saho, Tigre and minority Afar ethnic group. With a temperate climate, Leayten has no health facility but is nearer to a nearby town facility, Nefasit health station.

### Study design and study population

This study used cross-sectional analytical study design. The study population was women of child bearing age [15–49 years], who had at least one birth in the last 1–2 years by the time of data collection, (August–September 2017).

### Sampling design and sampling method

The respondents were obtained from three subzones, namely, Emni Hayli, Mai Aini, and Nefasit. These villages were randomly selected from a list of subzones with high proportion of home deliveries in the country. Within the selected subzones, the randomly selected villages were Hadish Adi, Serea, Genseba, Kelay Bealtat, Dirko, Mai Leham, Kudo Abour, Adi Koho, and Leayten. In the villages, census sampling was employed to interview all eligible women of age 15–49 years who gave at least one birth within the 2 years preceding the survey. Women who lived in the village for less than 6 months, who were severely ill, never gave at least one child birth, and women aged < 15 were excluded from the study.

### Conceptual framework and study variables

The conceptual framework for this study was constructed based on the three delays model of maternal healthcare utilization developed by Thaddeus and Maine [45] and later expanded by Gabrysch and Campbell to conceptually distinguish emergency care-seeking and preventive care-seeking behavior [7]. In our study, the adapted conceptual framework captures factors associated with the choice of delivery place in terms of the first delay in seeking care (predisposing characteristics), the second delay in identifying and reaching health facility (enabling characteristics), and the third delay in receiving quality care in health facility (perceived benefits and needs).

**Fig. 1:**
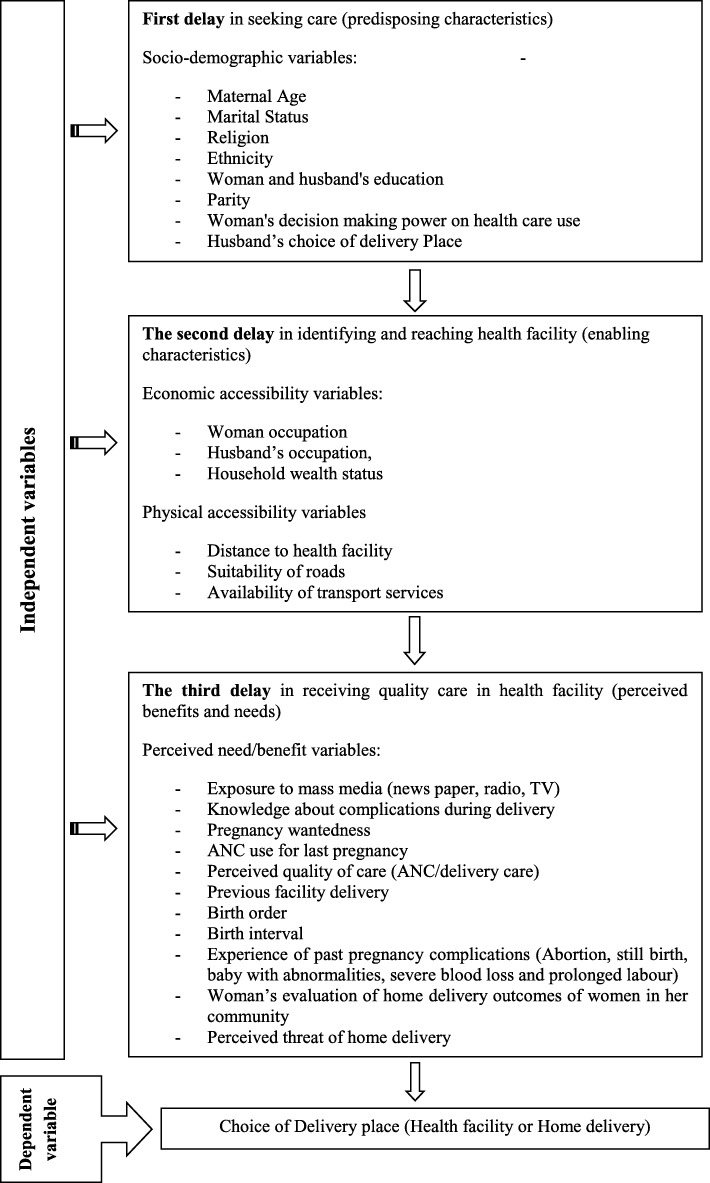
Conceptual framework for the determinants of choice of delivery place

The variables used to conceptualize the first delay were socio demographic factors such as maternal age, marital status, religion, ethnicity, parity, woman’s decision-making power on healthcare use, husband’s choice of delivery place, and woman and husband’s education. These predisposing factors are socio-cultural characteristics of individuals that exist prior to their illness that affect individual’s access to and use of health services. The second delay, often explained as logistical aspects of obtaining care, was framed as economic accessibility (woman and husband’s occupation, household wealth status, and cost for transport) and physical accessibility (distance to health facility, suitability of roads, and availability of transport services). The third delay, defined as a functional and health problems factors that generate the need for health care services, was represented by exposure to mass media, knowledge about complications during delivery, pregnancy wantedness, ANC use for last pregnancy, perceived quality of care (ANC/delivery care), previous facility delivery, birth order, birth interval, experience of past pregnancy complications, respondents’ evaluation of delivery outcomes of women in her community, and perceived threat of home delivery. The conceptual framework of the study is shown on Fig. 1.

### Data collection method

Structured questionnaire with open- and close-ended questions was employed to interview the respondents. The English version of the questionnaire was translated into a local language (Tigrigna) to ensure the respondents understand the contents properly. During data collection, filled questionnaires were counter checked for accuracy and completeness.

### Data processing and analysis

The data were analyzed using SPSS version 20 statistical software. The result is presented in frequency tables and proportions while chi-square test was used to determine statistically significant association between the variables. Bivariate and multivariate logistic regression analysis was then conducted to identify factors that affect the choice of delivery place. Results were presented in odds ratios with 95% confidence intervals with *p* < 0.05 taken as statically significant association. Performance of the regression model was assessed using the Nagelkereke coefficient or coefficient of determination (*R*^2^). *R*^2^ gives a picture of how the selected independent variables in the model adequately explain the variability of the outcome variable, in this case the choice of delivery place.

## Results

### Socio-demographic characteristics of the respondents

The mean age of the respondents was 28.56 ± 5.97. More than half (53.7%) of mothers were in the age range of 26–35 years. From the respondents, 90.6% were married, 85.7% were orthodox Christians, 88.9% belong to the Tigrigna ethnic group, and 42.7% reached primary school. Concerning to the occupational status of the respondent’s husband, more than half (62.2%) of them were governmental workers while majority of them were moderately educated. Final decision-making power on delivery place rests mainly on the mother (79.3%) while husband’s choice of delivery place was predominantly in health facilities (85.4%). Detailed socio-demographic information is shown in Table 1.

**Table 1:** Socio-demographic characteristics of the respondents (*n* = 309)

Variable	Category	*n* (%)	*p* value (*χ*^*2*^ test)
Maternal age	< 18	6 (1.9)	0.656
	19–25	98 (31.7)	
	26–35	166 (53.7)	
	35+	39 (12.6)	
Marital status	Married	280 (90.6)	0.183
	Single	12 (3.9)	
	Separated	17 (5.5)	
Religion	Orthodox	265 (85.8)	0.823
	Catholic	1 (0.3)	
	Muslim	43 (13.9)	
Ethnicity	Tigrigna	275 (89)	0.001
	Saho	31 (10.3)	
	Tigre	4 (1.3)	
Mother’s education	Illiterate	41 (13.3)	0.335
	Primary	132 (42.7)	
	Junior	87 (28.2)	
	Secondary	44 (14.2)	
	Diploma and above	5 (1.6)	
Husband’s education	Illiterate	37 (12.4)	0.008
	Primary	82 (27.4)	
	Junior	84 (28.1)	
	Secondary	74 (24.7)	
	Diploma and above	22 (7.4)	
Parity	< 2	112 (36.2)	0.454
	3–4	94 (30.4)	
	5–6	71 (23)	
	7+	32 (10.4)	
Final decision-making power on delivery place	Mother	245 (79.3)	0.001
	Mother and husband	51 (16.5)	
	Husband only	8 (2.6)	
	Other	5 (1.6)	
Husband’s choice of delivery place	Health facility	257 (85.4)	0.001
	Home delivery	44 (14.6)	

Table 2 shows factors related to the economic and physical availability identifying and reaching health facility. The majority of the women were housewives (86.7%), more than half (62.2%) of husband’s were government employees and half of the households had medium wealth status. While distance from health facility tends to be similar, the means of transport to health facility varied from as few as 1.4% using private vehicles to almost a third quarter of the women travel on foot to reach health facility.

**Table 2:** Economic and physical accessibility variables related to identifying and reaching health facility

Variable	Category	*n* (%)	*p* value (*χ*^*2*^ test)
Woman’s occupation	House wife	268 (86.7)	0.001
	Governmental worker	29 (9.4)	
	Merchant	5 (1.6)	
	Farmer	4 (1.3)	
	Daily worker	3 (1)	
Husband’s occupation	Governmental worker	186 (62.2)	0.010
	Merchant	9 (3)	
	Farmer	74 (24.7)	
	Daily worker	7 (2.3)	
	Students	3 (1)	
	Other	18 (6)	
Household wealth status^a^	Poor	123 (39.9)	0.041
	Medium	165 (53.6)	
	Rich	20 (6.5)	
Distance to health facility	< 2 km	25 (8.1)	0.001
	2–5 km	80 (25.9)	
	5–10 km	107 (34.6)	
	> 10 km	97 (31.4)	
Means of transport to health facility	On foot	211 (72.3)	0.001
	Traditional transport (mule, horse, karieza)	12 (4.1)	
	Public transport	64 (21.9)	
	Ambulance	1 (0.3)	
	Private vehicle	4 (1.4)	
Suitability of roads	Suitable	210 (68)	0.301
	Not suitable	99 (32)	

^a^Household wealth index is constructed using principal components analysis using financial indices (monthly income and expenditures) and asset indices (type of toilet, radio, TV, mobile telephone, fixed telephone, refrigerator, cattle, camels, horse, donkeys, or mules, goats, sheep, chicken, animal-drawn cart, bicycle, motorcycle, and car\truck)

Table 3 shows factors that are related to the perceived need/benefit for receiving quality care. Newspapers and television were largely inaccessible to most women compared to radio. Almost half (58.9%) of the respondents were not knowledgeable about complications during delivery. Antenatal care use was almost universal (92.2%) with 85.6% of the respondents judging the services as satisfactory. More than half (59.4%) of the respondents had no history of previous facility delivery while almost half of them (42.4%) had medium child-spacing practices. More than a quarter (36.6%) of the respondents had experience of past pregnancy complications, 40.5% have negative perception on the delivery outcomes of women in their community, but only 35.9% of the respondents see no threat in delivering at home.

**Table 3:** Variables related to the perceived need/benefit for receiving quality care

Variable	Category	*n* (%)	*p* value (*χ*^*2*^ test)
Exposure to mass media			
Reading newspapers	Daily	8(2.6)	0.017
	Sometimes	75(24.3)	
	Never	226(73.1)	
Listening radio	Daily	48(15.5)	0.219
	Sometimes	144(46.6)	
	Never	117(37.9)	
Watching TV	Daily	13(4.2)	0.524
	Sometimes	27(8.7)	
	Never	269(87.1)	
Knowledge about complications during delivery^a^	Knowledgeable	127 (41.1)	0.001
	Not knowledgeable	182 (58.9)	
Pregnancy wantedness	Yes	268 (86.7)	0.332
	No	41 (13.3)	
ANC use for last pregnancy	Yes	285 (92.2)	0.656
	No	24 (7.8)	
Perceived quality of care (ANC/delivery care)	Satisfactory	255 (85.6)	0.011
	Not satisfactory	43 (14.9)	
Previous facility delivery	Yes	125 (40.6)	0.001
	No	183 (59.4)	
Birth order of last pregnancy	First	55(17.8)	0.117
	Second	55(17.8)	
	Third	50(16.2)	
	Fourth	49(15.9)	
	Fifth	45(14.6)	
	More than six	55 (17.8)	
Birth interval^b^	Close spacing	120 (38.8)	0.004
	Medium	131 (42.4)	
	Distant	58 (18.8)	
Experience of past pregnancy complications^c^	Yes	113 (36.6)	0.379
	No	196 (63.4)	
Women’s perception of delivery outcomes of women in her community	Positive	115 (37.2)	0.001
	Negative	125 (40.5)	
	Mixed	69 (22.3)	
Perceived threats of home delivery	Threatening	198 (64.1)	0.001
	Not threatening	111 (35.9)	

^a^Respondents were asked to mention at least four complications that could happen during delivery and woman was categorized as not knowledgeable if she names less than four complications. If the respondents mention four or more complications, they were categorized as knowledgeable

^b^Close birth interval is defined as having deliveries at 1 year or less interval from last child, medium spacing from 1 year to 2 years, and birth spacing more than 3 years or above was categorized as having distant birth spacing

^c^Experience of abortion, still birth, baby with abnormalities, severe blood loss, and prolonged labor during last pregnancy

Regarding the choice of delivery place, 233 (75.4%) delivered their last child at home while 76 (24.6%) delivered their last child in health facilities. Table 4 details the variables regressed to determine factors associated with respondent’s choice of delivery place. In the bivariate regression analysis, use of health facility for childbirth increases with joint husband and mother decision-making in choosing on where to deliver, in households where husbands choose their partners to deliver in health facility and in households with medium wealth status.

**Table 4:** Bivariate and multivariate logistic regression analysis of factors associated with the choice of delivery place

Variable	Category	COR [95% CI]	AOR [95% CI]
Husband’s education	No education	0.08 [0.01–0.43]**	0.02 [0.01–0.54]*
	Primary	0.35 [0.12–0.96]*	0.61 [0.12–3.19]
	Junior	0.64 [0.24–1.70]	1.13 [0.22–5.84]
	Secondary	0.43 [0.15–1.18]	0.32 [0.06–1.73]
	Diploma and above®	1	1
Final decision making power on delivery place	Mother®	1	1
	Husband only	3.94 [2.09–7.43]	2.78 [0.36–21.09]
	Mother and husband	2.46 [0.56–10.66]**	5.42 [1.78–16.49]**
Husband’s choice of delivery place	Health facility	15.78 [2.13–6.81]**	2.32 [1.24–5.11]*
	Home delivery	1	1
Household wealth status	Poor	1	1
	Medium	2.05 [1.15–3.65]*	3.78 [1.38–10.37]*
	Rich	2.08 [0.71–6.04]	1.77 [0.31–10.09]
Distance to health facility	< 2 km	9.63 [3.44–26.90]**	14.67 [2.30–93.45]**
	2–5 km	1.01 [0.51–2.01]	1.03 [0.31–3.39]
	5–10 km	0.42 [0.20–0.88]*	0.39 [0.11–1.35]
	> 10 km®	1	1
Means of transport to health facility	On foot®	1	1
	Traditional transport (mule, horse, karieza)	9.17 [2.39–35.16]**	9.78 [1.23–77.26]*
	Public transport	0.77 [0.39–1.54]	1.19 [0.33–4.17]
	Ambulance	0.32 [0.23–1.23]	0.21 [0.11–1.12]
	Private vehicle	3.05 [0.420–22.25]	2.06 [0.25–1.11]
Reading news papers	Daily	2.34 [0.541–10.18]	3.77 [1.12–4.04]*
	Sometimes	2.20 [1.24–3.90]**	2.95 [1.01–8.59]*
	Never®	1	1
Knowledge about complications during delivery	Knowledgeable	4.95 [2.82–8.68]**	4.39 [1.63–11.83]**
	Not knowledgeable®	1	1
Perceived quality of delivery care	Satisfactory	3.68 [1.27–10.70]*	9.52 [1.91–47.50]**
	Not satisfactory®	1	1
Previous facility delivery	Yes	2.56 [1.51–4.35]**	2.69 [0.94–7.68]*
	No ®	1	1
Birth interval	Medium	1.68 [0.90–3.13]	2.20 [0.77–6.26]
	Distant	3.28 [1.61–6.69]**	11.92 [3.42–41.49]**
	Close spacing ®	1	1
Women’s perception of delivery outcomes of women in her community	Positive	0.50 [0.20–1.21]	1.59 [0.35–7.27]
	Negative	3.49 [1.70–7.15]**	1.31 [1.06–4.96]*
	Mixed ®	1	1
Perceived threats of home delivery	Threatening	7.94 [3.50–18.03]**	1.84 [1.46–3.38]*
	Not threatening ®	1	1

*COR* crude odds ratio, *AOR* adjusted odds ratio, *CI* confidence interval, *R* reference category, *p < 0.05, **p < 0.001

Short distance to reach health facility, using traditional transport systems, reading newspapers, and increased knowledge about complications during delivery were also found to increase the odds of delivering in health facility as were higher perceived satisfaction in the quality of care, past facility delivery experience, distant child spacing practices, negative evaluation of delivery outcomes women in their community, and perceiving home delivery as life threatening. Also, how a woman evaluates the outcomes of delivery in her community and her personal attitudes of delivering at home were important determinants on the choice of delivery place.

In the multivariate regression model, women whose husband’s had no formal education were less likely [adjusted odds ratio (AOR) = 0.02; 95% CI 0.01–0.54] to deliver in health facility. Respondents who reported joint woman and husband decision-making on place of delivery were more than five times more likely [AOR = 5.42; 95% CI 1.78–16.49] to deliver in health facility than women who make independent decision or where only husbands choose the delivery place. Also, respondents who report that their husbands choose health facility delivery were two times more likely [AOR = 2.32; 95% CI 1.24–5.11)] to deliver in health facility.

Households with medium wealth status, with a short distance to reach health facility, and use traditional transport systems were 3 [AOR = 3.78; 95% CI 1.38–10.37], 14 [AOR = 14.67; 95% CI; 2.30–93.45], and 9 times [AOR = 9.78; 95% CI 1.23–77.26] more likely to deliver in health facility respectively. Respondents who read newspaper daily or infrequently had three [AOR = 3.77; 95% CI 1.12–4.04] and almost three times [AOR = 2.95; 95% CI 1.01–8.59] higher odds of delivering in health facility respectively. Similarly, women who had higher knowledge about complications during delivery were four times more likely [AOR = 4.39; 95% CI 1.63–11.83] to deliver in health facility.

Respondents who reported higher satisfaction in the quality of care were nine times more likely [AOR = 9.52; 95% CI 1.91–47.50] to delivery in health facility. Previous experience of facility delivery had a double likelihood of health facility delivery [AOR = 2.69; 95% CI 0.94–7.68] while women who had distant child spacing practices were 11 times more likely [AOR = 11.92; 95% CI 3.42–41.49] to have facility delivery. Women who evaluate the outcomes of delivery in her community as negative [AOR = 1.31; 95% CI 1.00–4.96] and women who think that delivering at home is life threatening [AOR = 1.84; 95% CI 1.46–3.38] were more likely to deliver in health facility. Overall, the constructed regression model performed well as the coefficient of determination (Nagelkerke *R*^2^) was 71%, indicating the selected variables explained about 70% of the variability on the choice of delivery place.

## Discussion

This study aimed to assess factors influencing the choice of delivery place among mothers living in rural communities of Eritrea. Overall, the proportion of women who had health facility delivery was only 24.6%. This figure is much lower than the most recent national LQAS survey that reports 62% of mothers deliver their child in health facilities [39]. In our study, the village level of health facility delivery ranged from 32.4% in Mai Aini Village, 19.3% in Leayten Village, and 16.9% in Emni Hayli. In light of this, our finding questions the implicit assumption of taking national, zonal, or district averages as representative of most communities and application of these figures to policy formulation and budget allocation. Simply put, every activities or interventions that targets specific communities, particularly when dealing with urban/rural differences, should not under estimate the level of inequities even in communities located in close geographical areas.

While the proportion of mothers who had health facility delivery was significantly lower than home births, the primary objective of this study was to explore the range of factors that are influential in determining the choice delivery place. These factors are below discussed in the context of the three delays model.

In light of the first delay (predisposing factors), several variables were found to be independent predictors of the choice of delivery place. These variables reflect the socio-cultural context in which women live and how these factors affect their preferences in access to and use of health services. Studies have showed that higher level of husband’s education is associated with greater knowledge and increased risk perception of home delivery. Higher spouse education is also related to modern attitudes to health facilities, better communication with partners and lending higher degree of autonomy to their wives and hence facilitates health facility use. In this regard, a number of systematic reviews on determinants of health facility delivery have shown that husband’s education consistently increases health facility use, but often with smaller effect than the mother’s education [7, 46–48]. Similarly, this study found that women whose husbands have no or little education are less likely to deliver in health facilities. However, mother’s educational level was not associated with the place of delivery. Although studies report that mother’s education, employment, and higher economic status are related with the choice of delivery place, some studies in Eritrea have shown that reproductive preferences tend to have less relationship with these variables [36, 49]. These studies argue that, in a largely patriarchal society like in Eritrea, reproductive health preferences and practices are not solely determined by women characteristics. This culturally molded, male-dominated power differential between husbands and wives may explain why even women with higher educational level may fail to translate their choice of delivery place into actual behavior, if their husbands are opposed to their choice. This study also supports this notion as the majority of women who had junior or secondary level of education, but their husbands choose their wives to deliver at home predominantly had home delivery in both categories. This was also true for mother’s employment. Thus, due to the observed husband’s influence on facility delivery, our findings suggest that interventions that encourage males to have more formal education should be given due attention to divert husband’s power and influence to support facility delivery.

Final decision-making power in choosing the place of delivery is widely recognized as an important predictor of health facility delivery [50, 51]. Intra-familial decision-making power regarding the use of maternal healthcare services is strongly influenced by the values and opinions of husbands, mothers-in-law, close relatives, traditional birth attendants, and other community members. Hence, the use of maternal health services, including facility delivery can be seriously undermined by women’s lack of decision-making autonomy through complex processes of gender inequality [32, 52]. For example, in their multi country analysis on the delivery place of poor women in developing countries, Montagu and his colleagues report that the most common reason given by both the poorest and richest women for not delivering in a facility was that it was deemed “not necessary” by a household decision-maker in more than half of the respondents. The authors have explained the motivation for delivering at home as to be influenced by social and cultural beliefs at the household and community levels [53].

In case of Eritrea, women who live in rural areas have limited decision-making power and hence little control over their reproductive health decisions. Previous studies in Eritrea have shown that regardless of the variations in women’s autonomy, joint decision-making or spousal communication rather than independent decision-making is a significant predictor of reproductive health behavior [49, 54]. In our study, respondents that reported joint mother and husband decision-making were five times more likely to deliver in health facility. Joint decision-making power increased with increasing educational level of the mother, suggesting higher educational attainment can have positive impact on institutional delivery. In line with this viewpoint, close analysis of our finding also shows women whose husbands are more educated tend to report more joint decision-making on the place of delivery. Assuming that educated husbands have more propensities for better communication skills and include wives in deciding on where to deliver, our findings suggest that targeting male involvement in health education and awareness campaigns could have a tangible result in rural settings of Eritrea. The pathway of these interventions could be by directly influencing husband’s attitudes that favor institutional delivery or indirectly by creating an easier negotiation environment for their wives to put their opinion on reproductive health decisions of the household.

In many studies, male involvement in the use of maternal health services has been increasingly recognized as equally important factor as direct factors related to women themselves [7, 19, 23, 29]. In this study, husband’s choice of delivery place was found to have independent predictive power on the choice of delivery place. Respondents whose husbands choose health facility delivery were two times at odds to deliver in health facilities. This finding is similar to studies conducted in developing countries [55, 56]. As previously explained, this is particularly important in Eritrea as husbands tend to have more say on major household decisions, including decisions on where their wives should deliver their babies. [10, 54]. It is important to note, however, that husband’s choice of delivery was not associated with their occupation or educational level. This implies that efforts to change husband’s attitude in favor of health facility delivery may be more complicated than one might expect. This could be because of the hidden but potentially potent culturally guaranteed male dominance irrespective of his educational status or occupation is still on strong hold in rural Eritrean communities. To further analyze this issue, further studies on the socio-cultural determinants of reproductive health practices in general and health facility delivery in particular seems timely before initiating any action to curb the apparently negative influence of socio-cultural factors on health facility delivery.

In the perspective of the second delay, both economic and physical accessibility to health facilities were found to have significant influence on women’s preference of delivery place. Many studies have noted that women from poor households are less likely to use delivery services as facility-based delivery causes financial hardship and challenges families to pay even for nominal fees, transport fees, and compensating the gap left at home to care for children. Some studies have also reported that poor women tend to face violation in their dignity and experience abusive treatment from the health personnel during facility deliveries [57, 58].

In this study, households with medium wealth status had higher odds of delivering in health facility. In Eritrea, the issue of financial expenses for facility delivery services tends to be minimal as the health care services are largely subsided by the government where service users are required to pay only nominal user fees [59]. Even so, households with limited financial capacity are unable to pay for transport in case of referral or where the health facility is far from home. This finding is consistent with results of a study conducted by the World Bank. The study reports that, in Eritrea, travel to a referral facility on public or private transportation, patients have to pay on an average $26 from a referral hospital; $17 from a community hospital, and $32 from a health center/station. This cost of transport represents a fairly large proportion of the annual GDP per capita in Eritrea ($336) [60]. Based on this, as the studied villages predominantly use health stations or health centers, referral cases have to pay the highest fair for transport to their nearest higher health institution. Considering that women who live in rural areas have no income of their own and their husbands largely have inadequate income, expenses associated with institutional delivery amounts to unbearable payment plans for transport to reach health facilities and making arrangements for other related items like bed sheet, blankets, hot drinks, food stuff for the mother, and the person accompanying her. Similarly, the EPHS 2010 showed that 39% of the respondents mentioned financial constraints as the most common barrier to using health facility for child birth [38]. This shows that for the past two decades, financial difficulties have been and continue to be a major threat in seeking health service delivery. It is also apparent that a concerted effort needs to be made for making health services not only physically reachable but economically accessible to all women of Eritrea. To attain this, incentives or compensations for transport expenses to alleviate the cost of transport in rural communities might have considerable impact in increasing health facility delivery.

Apart from financial constraints, many studies in developing countries have shown that distance to reach health facility has a strong effect on the choice of and access to health services [19, 29, 35]. In this study, distance to health facility was found to be an important predictor of health facility delivery. Women who reside within 2 km distance to health facility were 14 times more likely to deliver in health facility than women who have to travel for more than 2 km to reach the nearest health facility. This is not unexpected in view that the study areas are rural villages where distance is an impenetrable barrier for health service use irrespective of the mother, husband, or the household’s characteristics. As almost 80% of Eritrean population live in rural areas, proximity to health facility is likely to affect use of the available services. Distance has a direct impact on the choice of delivery place as transportation to health facilities is usually unavailable or costs more than what is affordable. The average distance to a primary health care facility for many rural communities in Eritrea is from 10 to 18 km, and not all health stations are equipped to provide delivery care. The referral support capacity is weak as there is lack of communication links between various levels of the system and transportation of emergency cases is not guaranteed. Our finding is strikingly similar with the EPHS 2010 which found that 33% of the respondents mentioned distance to health facility as one of the common reasons or barriers for not seeking delivery service [38].

Regarding the means of transport to reach health facility, this study found that women who use traditional transport systems like mule, horse, and karieza were more likely to deliver in health facility. This is again similar with the EPHS 2010 which found that 35% of the respondents complain having to take transportation as a main barrier in using health facility [38]. Thus, to positively affect women who need to be moved to the next level of service, sustainable solutions should be explored to improve transport systems to address the common barrier of lack of transport at the community level. For instance, there are currently many maternity waiting homes scattered across the whole country [61]. The expectation is that these homes will address the problems mothers experience reaching a health facility at delivery time. After analyzing their cultural feasibility, cost, and efficacy, these initiatives could be replicated in remote communities of Eritrea.

In variables that capture the third delay of seeking care (women’s perceived need and benefits of delivering in health facilities), several factors were found to have independent predictive power on the choice of delivery place. Exposure to information on television, radio, and in the print media can increase knowledge and awareness of new ideas, social changes, and can affect an individual’s perceptions and behavior, including those about health matters. As such, the utilization of the mass media to influence women’s knowledge about delivery risks, availability of services and promoting the benefits of health facility delivery has been employed as a major strategy in many countries [7, 23, 26, 30]. However, in many studies, the effect of information availability about the risks posed by childbirth in radio, TV, or newspapers on reproductive health behaviors have been inconsistent [62, 63]. Some studies report an association with increased use of facilities for delivery but not in others. In this study, only reading newspaper was found to be linked with higher odds of delivering at health facility. The possible explanation for this could be mothers who read newspapers are more educated and hence more receptive to the frequently delivered health message on different maternal and child health-related information and the importance of institutional delivery.

Knowledge about complications during delivery has been recognized as a predictor of health facility delivery in case complications arise [64–66]. In this study, respondents who were knowledgeable about the complications that may arise during delivery were four times more likely to have health facility delivery than women who do not have adequate knowledge. Knowledge about complication increased with increasing maternal age but was considerably less so in mothers above 35 years of age. This pattern was also seen in health facility delivery, where mothers who were aware of obstetric complications had more facility delivery as were women who reported to have attended at least one antenatal care. Past experiences of obstetric complications was not associated with the level of knowledge, suggesting that the reported knowledge largely might be from exposure to health information gained while utilizing health services. This can also be an indication that other reproductive health services like antenatal care can be used as an opportunity to inform women about the benefits of institutional deliveries.

Individual perception of quality care has an important bearing on the choice of service preferences [45]. These perceptions stem from personal experiences or experiences of others or in some cases, merely from public opinion. Often, studies have reported that individual perception on the quality of services tend to be influenced much more by time given for the patient, waiting time, and the level of health provider interpersonal communication skills than actual measurable quality of service indicators such as facility set up, equipment, and staffing [67].

In this study, perceived quality of care was an important independent predictor of delivery place. Respondents who report satisfactory level of service quality were nine times more likely to deliver in health facility. In their study on the quality of the maternal health system in Eritrea, Sharan M. and her colleagues have noted insufficient infrastructure and long waiting times due to shortage of health workers affects the quality of care and maternal health outcome in Eritrea [68]. Similarly, Kifle et al. found that mothers who were not satisfied with the general hospital delivery practices in Eritrea were the least satisfied with the way health professionals communicated with them and the level of cleanliness of the delivery environment [69].

In many studies, previous facility delivery has been a consistent predictor of facility delivery [19, 46–48]. The suggested pathways of relationship also tend to be similar in many cases. Women who delivered in health facilities are more likely to continue to deliver in health facilities, irrespective of other confounding variables. This is particularly more plausible when mothers had complicated delivery, are familiar with the rendered services, or perceive the services as satisfactory. In this study, previous facility delivery was associated with higher odds of delivering in health facility. Of note, irrespective of their past health service delivery experience, young mothers had more facility delivery. But for the older mothers, even if they had past health facility experience, they generally preferred home delivery. This could be due to the already developed “confidence,” attitudes that delivery is a natural process and does not need assistance from healthcare provider or the persistence of the perception that modern healthcare is not deemed necessary with increasing mothers experience or knowledge accumulated from previous pregnancies and births.

In this study, birth interval rather than birth order was found to have independent predictive power on the choice of delivery place. Compared to women who had close birth spacing, mothers who had relatively distant birth interval practices were 11 times more likely to deliver in health facilities. This finding is similar with studies conducted in developing countries [19]. Historically, the level of unmet need for modern contraception has been very high in Eritrea. Studies have shown that the most important reason for unmet need is lack of knowledge of methods or of a source of supply. In Eritrea, currently married women with higher parity, low autonomy, low or medium household economic status, and who know no method of contraception or source of supply are identified as the most likely group to have an unmet need [38, 70]. Hence, our findings calls for an increased family planning awareness campaigns for both mothers and husbands to have a reasonable child spacing practices.

Studies that analyze the effect of contextual factors or community influences on the choice of delivery place have documented that individual reproductive attitudes or actual decisions are strongly influenced by how other members of her community commonly deal with facility delivery. Also, how her decision will be judged by her immediate community has an important influence on individual decision-making [18, 24]. In this study, respondents who reported negative home birth outcomes in their community were more likely to actually deliver in health facility than women who claimed that home delivery they have witnessed in their community had a positive experience. Similarly, how a woman perceives the potential benefits and outweighs the threats of home delivery may pose to the mother and the infant has important bearing on future choice of delivery place [7, 25, 27]. This was reflected in our findings as women who see home delivery as a threat were more likely to deliver in health facility. This perception was common among the young, the wealthy, and the more educated mothers. This implies that health education focusing on the potential threats of delivering at home to women of reproductive age in general and to the less educated and poor women in particular may be an important strategy to the continuous effort done to increase institutional delivery.

The results of this study have strengths and limitations. The reliability of the data was maintained as the study was community-based survey and same sex interviewers were used who were non-health workers and largely unaware of the desired answers. The census sampling technique and high response rate also add to the credibility of the findings. However, although the study has come up with important findings, the results should be interpreted with caution. First, the cross-sectional nature of the study prevents elucidating the direction of the cause and effect relationship of the independent variables with the choice of delivery place. The possibility of recall bias might also have influence in misreporting actual events. The study setting could also be significantly different contextually with other rural parts of the country, particularly in zones with larger population, diverse ethnic distribution and with more number of available health services. This contextual difference might warrant further studies in different geographical areas and in diverse communities of the country.

## Conclusion

The level of health facility delivery in the selected rural communities was significantly lower than the national average. Women whose husbands are educated, who practice joint decision-making with husbands on delivery place, and women whose husbands choose health facility delivery were more likely to have health facility delivery. Respondents who had medium wealth status, have access to health facility within 2 km distance, and women with traditional means of transport were also more likely to deliver in health facility. Similarly, women who read newspapers, which have knowledge about complications during delivery, good perception on the quality of care they received, had previous facility delivery, have negative experiences of delivery outcomes in her community, and women who perceive home delivery as life threatening were more likely to deliver in health facility.

To increase facility delivery, the study findings suggest raising women’s awareness on the benefits of delivering in health facility, male involvement in the use of maternal health services, increasing women decision-making power, and incentives or compensations for transport expenses to alleviate the cost of transport could prove effective. Also, this study identified that efforts to shorten distance to reach health facility and addressing common barriers of lack of transport and health education focusing on the potential threats of delivering at home at the individual and community level can have significant contribution to increase health facility delivery.

## Abbreviations

ANC: Antenatal care
AOR: Adjusted odds ratio
EPHS: Eritrean Population and Health Survey
HSSDP: Health Sector Strategic Development Plan
LQAS: National lot quality assurance survey
MMR: Maternal mortality ratio
SBA: Skilled birth attendant
SSA: Sub-Saharan Africa
WHO: World Health Organization

## References

[1] Trends in maternal mortality. 1990 to 2015: estimates by WHO, UNICEF, UNFPA, World Bank Group and the United Nations Population Division. ISBN 978 92 4 1565141. Available at https://openknowledge.worldbank.org/bitstream/handle/10986/23550/report.pdf;sequence=1.

[2] Graham WJ, Fitzmaurice AE, Bell JS, Cairns JA (2004). The familial technique for linking maternal death with poverty. Lancet.

[3] Alvarez JL, Gil R, Hernandez V, Gil A (2009). Factors associated with maternal mortality in Sub-Saharan Africa: an ecological study. BMC Public Health.

[4] Mills A (2014). Health care systems in low- and middle-income countries. N Engl J Med.

[5] Bhutta ZA, Black RE (2013). Global maternal, newborn, and child health—so near and yet so far. N Engl J Med.

[6] Ronsmans C, Graham WJ (2006). Maternal mortality: who, when, where, and why. Lancet.

[7] Gabrysch S, Campbell OM (2009). Still too far to walk: literature review of the determinants of delivery service use. BMC Pregnancy Childbirth.

[8] Campbell OM, Graham WJ (2006). Strategies for reducing maternal mortality: getting on with what works. Lancet.

[9] WHO (2004). Making pregnancy safer: the critical role of the skilled attendant.

[10] Khan KS, Wojdyla D, Say L, Gulmezoglu AM, Van Look PF (2006). WHO analysis of causes of maternal death: a systematic review. Lancet.

[11] Stanton C, Blanc AK, Croft T (2007). Skilled care at birth in the developing world: progress to date and strategies for expanding coverage. J Biosoc Sci.

[12] Bloom SS, Lippeveld T, Wypij D (1999). Does antenatal care make a difference to safe delivery? A study in urban Uttar Pradesh, India 8894. Health Policy Plan.

[13] Yanagisawa S, Oum S, Wakai S (2006). Determinants of skilled birth attendance in rural Cambodia 90 96. Tropical Med Int Health.

[14] Buor D, Bream K (2004). An analysis of the determinants of maternal mortality in sub-Saharan Africa. J Women's Health.

[15] Gabrysch Sabine, Cousens Simon, Cox Jonathan, Campbell Oona M. R. (2011). The Influence of Distance and Level of Care on Delivery Place in Rural Zambia: A Study of Linked National Data in a Geographic Information System. PLoS Medicine.

[16] Singh Kavita, Brodish Paul, Suchindran Chirayath (2014). A Regional Multilevel Analysis: Can Skilled Birth Attendants Uniformly Decrease Neonatal Mortality?. Maternal and Child Health Journal.

[17] Lawn JE, Cousens S, Zupan J (2005). 4 million neonatal deaths: when? Where? Why?. Lancet.

[18] Stephenson R, Baschieri A, Clements S, Hennink M, Madise N (2006). Contextual influences on the use of health facilities for childbirth in Africa. Am J Public Health.

[19] Moyer CA, Mustafa A (2013). Drivers and deterrents of facility delivery in sub-Saharan Africa: a systematic review. Reprod Health.

[20] Letamo G, Rakgoasi SD (2003). Factors associated with non-use of maternal health services in Botswana. J Health Popul Nutr.

[21] Onah HE, Ikeako LC, Iloabachie GC (2006). Factors associated with the use of maternity services in Enugu, southeastern Nigeria. Soc Sci Med.

[22] Stekelenburg J, Kyanamina S, Mukelabai M, Wolffers I, van Roosmalen J (2004). Waiting too long: low use of maternal health services in Kalabo, Zambia. Tropical Med Int Health.

[23] Shifraw T, Berhane Y, Gulema H, Tamil Kendall T, Austin A (2016). A qualitative study on factors that influence women’s choice of delivery in health facilities in Addis Ababa, Ethiopia. BMC Pregnancy and Childbirth.

[24] YADAV AWDHESH, KESARWANI RANJANA (2015). EFFECT OF INDIVIDUAL AND COMMUNITY FACTORS ON MATERNAL HEALTH CARE SERVICE USE IN INDIA: A MULTILEVEL APPROACH. Journal of Biosocial Science.

[25] Aremu O, Lawoko S, Dalal K (2011). Neighborhood socioeconomic disadvantage, individual wealth status and patterns of delivery care utilization in Nigeria: a multilevel discrete choice analysis. Int J Women's Health.

[26] Bell J, Curtis SL, Alayón S: Trends in delivery care in six countries. DHS Analytical Studies No 7 2003. ORC Macro and International Research Partnership for Skilled Attendance for Everyone (SAFE). Calverton, Maryland USA). [http://www.meas.uredhs.com/pubs/pub_details.cfm? ID=482&srchTp=advanced]

[27] Mekonnen ZA, Lerebo WT, Gebrehiwot TG, Abadura SA (2015). Multilevel analysis of individual and community level factors associated with institutional delivery in Ethiopia. BMC Res Notes.

[28] Agadjanian V, Yao J, Hayford SR (2016). Place, time and experience: barriers to universalization of institutional child delivery in rural Mozambique. Int Perspect Sex Reprod Health.

[29] Bohren MA, Hunter EC, Munthe-Kaas HM, Souza JP, Vogel JP, Gülmezoglu AM (2014). Facilitators and barriers to facility-based delivery in low- and middle-income countries: a qualitative evidence synthesis. Reprod Health.

[30] Amponsah E, Moses I (2009). Expectant mothers and the demand for institutional delivery: do household income and access to health information matter? Some insight from Ghana. Eur J Soc Sci.

[31] Kusuma Yadlapalli Sriparvati, Kaushal Sonia, Garg Rishi, Babu Bontha Veerraju (2018). Birth preparedness and determinants of birth place among migrants living in slums and slum-like pockets in Delhi, India. Sexual & Reproductive Healthcare.

[32] Speizer IS, Story WT, Singh K (2014). Factors associated with institutional delivery in Ghana: the role of decision-making autonomy and community norms. BMC Pregnancy Childbirth.

[33] Kruk ME, Prescott MR (2012). The role of health systems and policies in promoting safe delivery in low- and middle-income countries: a multilevel analysis. Am J Public Health.

[34] Anand S, Barnighausen T (2004). Human resources and health outcomes: cross-country econometric study. Lancet.

[35] Houweling TA, Ronsmans C, Campbell OM, Kunst AE (2007). Huge poor-rich inequalities in maternity care: an international comparative study of maternity and child care in developing countries. Bull World Health Organ.

[36] Favali L, Pateman R (2003). Blood, Land and Sex, Legal and Political Pluralism in Eritrea.

[37] World health statistics (2017). 2017: monitoring health for the SDGs, Sustainable Development Goals.

[38] The State of Eritrea National Statistics Office (NSO), Fafo Institute for Applied International Studies (AIS) (2013). Eritrea Population and Health Survey 2010. Eritrea population and health survey 2010.

[39] ERITREA – Lot Quality Assurance Sampling survey (LQAS) report on HIV/AIDS, TB, STI, Malaria and Maternal Health. Communicable Diseases Control Division, Ministry of Health, Eritrea, 2017. Available from the ministry of health head office.

[40] Araya Winta, Johnson-Mallard Versie, Evans Mary E, Beckstead Jason W, McNerney Diane, Shelton Melissa Molinari, Jevitt Cecilia (2012). Overview of maternal mortality in Eritrea, sub-Saharan Africa. African Journal of Midwifery and Women's Health.

[41] The second Health Sector Strategic Development Plan II (2017–2021). Ministry of Health, Asmara, Eritrea, 2017. Available at http://www.nationalplanningcycles.org/sites/default/files/planning_cycle_repository/eritrea/eritrea_hssdp_ii_21022017.pdf.

[42] Holzgreve W, Greiner D, Schwidtal P (2012). Maternal mortality in Eritrea: improvements associated with centralization of obstetric services. Int J Gynaecol Obstet.

[43] Turan JM, Tesfagiorghis M, Polan ML (2011). Evaluation of a community intervention for promotion of safe motherhood in Eritrea. J Midwifery Womens Health.

[44] Almedom A, Tesfamichael B, Saeed Z, Muller J, Masci C, Alemu Z (2005). ‘Hope’ makes sense in Eritrean sense of coherence, but ‘loser’ does not. J Loss Trauma.

[45] Thadeus S, Maine D (1994). Too far to walk: maternal mortality in context. Soc SciMed.

[46] Kyei-Nimakoh M, Carolan-Olah M, McCann TV (2017). Access barriers to obstetric care at health facilities in sub-Saharan Africa—a systematic review. Syst Rev.

[47] Jeffery Patricia, Jeffery Roger (2010). Only when the boat has started sinking: A maternal death in rural north India. Social Science & Medicine.

[48] Banke-Thomas OE, Banke-Thomas AO, Ameh CA (2017). Factors influencing utilisation of maternal health services by adolescent mothers in low-and middle-income countries: a systematic review. BMC Pregnancy Childbirth..

[49] Woldemicael G (2009). Women’s autonomy and reproductive preferences in Eritrea. J Biosoc Sci.

[50] Fotso JC, Ezeh A, Madise N, Ziraba A, Ogollah R (2009). What does access to maternal care mean among the urban poor? Factors associated with use of appropriate maternal health services in the slum settlements of Nairobi, Kenya. Matern Child Health J.

[51] Ahmed S, Creanga AA, Gillespie DG, Tsui AO (2010). Economic status, education and empowerment: implications for maternal health service utilization in developing countries. PLoS One.

[52] Ganle JK, Obeng B, Segbefia AY, Mwinyuri V, Yeboah JY, Baatiema L (2015). How intra-familial decision-making affects women’s access to, and use of maternal healthcare services in Ghana: a qualitative study. BMC Pregnancy Childbirth..

[53] Montagu D, Yamey G, Visconti A, Harding A, Yoong J (2011). Where do poor women in developing countries give birth? A multi-country analysis of demographic and health survey data. PLoS One.

[54] Woldemicael G (2010). Do women with higher autonomy seek more maternal health care? Evidence from Eritrea and Ethiopia. Health Care Women Int.

[55] Iftikhar Ul Husnain M, Rashid M, Shakoor U. Decision-making for birth location among women in Pakistan: evidence from national survey. BMC Pregnancy Childbirth. 2018;18(1):226. 10.1186/s12884-018-1844-8. 10.1186/s12884-018-1844-8 PMC6000961 29898695

[56] Nesbitt Robin C., Lohela Terhi J., Manu Alexander, Vesel Linda, Okyere Eunice, Edmond Karen, Owusu-Agyei Seth, Kirkwood Betty R., Gabrysch Sabine (2013). Quality along the Continuum: A Health Facility Assessment of Intrapartum and Postnatal Care in Ghana. PLoS ONE.

[57] Muzaffar N (2015). Maternal health and social determinants: a study in Jammu and Kashmir. Public Health Res.

[58] Bohren MA, Vogel JP, Hunter EC, Lutsiv O, Makh SK, Souza JP (2015). The mistreatment of women during childbirth in health facilities globally: a mixed-methods systematic review. PLoS Med.

[59] Habtom GebreMichael Kibreab, Ruys Pieter (2007). The choice of a health care provider in Eritrea. Health Policy.

[60] Sharan M, Ahmed S, Naimoli JF, Ghebrehiwet M, Rogo K. Health System Readiness to Meet Demand for Obstetric Care in Eritrea: Implications for Results-Based Financing (RBF). The World Bank Working Paper. 2010. Available: http://www.rbfhealth.org/system/files/eritrea%20maternal%20paper.pdf. Acessed 15 Aug 2018.

[61] Andemichael G, Haile B, Kosia A, Mufunda J. Maternity waiting homes: a panacea for maternal / neonatal conundrums in Eritrea. J Eritrean Med Assoc. 2009;4(1):18–21. 10.4314/jema.v4i1.52112.

[62] Ekukudo IW (2015). Information as a determinant of utilization of family planning services in rural Akwa Ibom state of south-south Nigeria. Mediterr J Soc Sci.

[63] Jah F, Connolly S, Barker K, Ryerson W (2014). Gender and reproductive outcomes: the effects of a radio serial drama in northern Nigeria. Int J Popul Res.

[64] Gabrysch Sabine, Zanger Philipp, Seneviratne Harshalal R., Mbewe Reuben, Campbell Oona M. R. (2011). Tracking progress towards safe motherhood: meeting the benchmark yet missing the goal? An appeal for better use of health-system output indicators with evidence from Zambia and Sri Lanka. Tropical Medicine & International Health.

[65] Phoxay Chandavone, Okumura Junko, Nakamura Yasuhide, Wakai Susumu (2001). Influence of Women's Knowledge on Maternal Health Care Utilization in Southern Laos. Asia Pacific Journal of Public Health.

[66] Gage AJ (2007). Barriers to the utilization of maternal health care in rural Mali. Soc Sci Med.

[67] Larson E, Hermosilla S, Kimweri A, Mbaruku GM, Kruk ME (2014). Determinants of perceived quality of obstetric care in rural Tanzania: a cross-sectional study. BMC Health Serv Res.

[68] Sharan M, Ahmed S, Ghebrehiwet M, Rogo K (2011). The quality of the maternal health system in Eritrea. Int J Gynecol Obstet.

[69] Kifle Meron Mehari, Ghirmai Filmon Abraham, Berhe Soliana Amanuel, Tesfay Winta Sium, Weldegebriel Yodit Teklemariam, Gebrehiwet Zebib Tesfamariam (2017). Predictors of Women’s Satisfaction with Hospital-Based Intrapartum Care in Asmara Public Hospitals, Eritrea. Obstetrics and Gynecology International.

[70] Woldemicael G (2011). Currently married women with an unmet need for contraception in Eritrea: profile and determinants. Can Stud Popul.

